# How do Medical Societies Select Science for Conference Presentation? How Should They?

**DOI:** 10.5811/westjem.2015.5.25518

**Published:** 2015-07-02

**Authors:** Thomas M. Kuczmarski, Ali S. Raja, Daniel J. Pallin

**Affiliations:** * Brigham and Women’s Hospital, Department of Emergency Medicine, Boston, Massachusetts; † Massachusetts General Hospital, Department of Emergency Medicine, Boston, Massachusetts; ‡ Harvard Medical School, Brigham and Women’s Hospital, Department of Emergency Medicine, Boston, Massachusetts

## Abstract

**Introduction:**

Nothing has been published to describe the practices of medical societies in choosing abstracts for presentations at their annual meetings. We surveyed medical societies to determine their practices, and also present a theoretical analysis of the topic.

**Methods:**

We contacted a convenience sample of large U.S. medical conferences, and determined their approach to choosing abstracts. We obtained information from web sites, telephone, and email. Our theoretical analysis compares values-based and empirical approaches for scoring system development.

**Results:**

We contacted 32 societies and obtained data on 28 (response rate 88%). We excluded one upon learning that research was not presented at its annual meeting, leaving 27 for analysis. Only 2 (7%) made their abstract scoring process available to submitters. Reviews were blinded in most societies (21;78%), and all but one asked reviewers to recuse themselves for conflict of interest (96%). All required ≥3 reviewers. Of the 24 providing information on how scores were generated, 21 (88%) reported using a single gestalt score, and three used a combined score created from pooled domain-specific sub-scores. We present a framework for societies to use in choosing abstracts, and demonstrate its application in the development of a new scoring system.

**Conclusions:**

Most medical societies use subjective, gestalt methods to select research for presentation at their annual meetings and do not disclose to submitters the details of how abstracts are chosen. We present a new scoring system that is transparent to submitters and reviewers alike with an accompanying statement of values and ground rules. We discuss the challenges faced in selecting abstracts for a large scientific meeting and share the values and practical considerations that undergird the new system.

## INTRODUCTION

Medical research is usually first shared publicly as a summary, called an “abstract,” presented at a scientific meeting. Abstract presentation is a crucial means by which the community exchanges information. Half of abstracts lead to formal publication.^1^–^2^

We became interested in abstract scoring during a planned update to the abstract scoring system of the Society for Academic Emergency Medicine. We began with a review of prior studies to determine how societies evaluated abstracts. Some prior research has evaluated scoring systems of individual societies according to inter-rater reliability. 3–7 Additional studies have investigated the validity and sensibility of specific scoring methods for individual societies.^3^,^8^ The value of blinding has been studied as well. 9 These approaches represent calibration of various measurement tools, and evaluation of individual scoring systems. But we were unable to identify any studies that compared procedures from one society to the next, or provided any descriptive overview of common practices.

This paper analyzes approaches to scoring abstracts for large medical conferences. The evaluation had an empirical component and a theoretical component. In the empirical portion, we surveyed a convenience sample of large U.S. medical societies to determine their approach to choosing abstracts for their annual meetings. In the theoretical portion of the study, we present a framework for understanding how a medical society might choose a scoring system. We present an example of how these empirical and theoretical considerations were used to develop a scoring system.

## METHODS

### Survey Methods

For the survey portion of the project, we contacted 32 medical societies, chosen at convenience and based on their attendance size. We chose the societies at convenience by reviewing a list of medical societies and choosing large societies that, in our opinion, would be relevant comparators. 10 We began with the list of the 50 largest medical conferences and eliminated ones that did not seem to be relevant comparators. For example, we included only academic medical conferences and excluded industrial conferences. We conducted a survey consisting of one qualitative and four quantitative data points: whether the scoring system was publicly available (vs. confidential), whether reviewers were blinded, whether reviewers could recuse themselves for conflicts of interest, the number of reviewers per abstract, and whether the final score represented a combined score created from pooled domain-specific sub-scores, or a single gestalt score. Study investigators chose these data points based on their pertinence and importance to the abstract scoring process. While there were many possible data to explore, we believe that when examined in aggregate, these data points provide a clear picture of how societies select abstracts at annual meetings.

We gathered data systematically. First, we examined each society’s annual meeting website. When data were unavailable via the internet, we contacted societies via telephone and email to collect all remaining information. These phone calls and emails were typically directed towards the director or associate director of operations for the society’s annual meetings. A specific telephone script or email template was not used, as we were merely trying to obtain the aforementioned five data points. We did not seek to infer population characteristics from the sample, and our sample was not random. Therefore, use of inferential statistics would not be appropriate, and we refrain from reporting confidence intervals or stochastic and inferential measures. We did not seek institutional review board review of this research project, as it was not human-subjects research. After data collection was complete, all surveyed societies were emailed this manuscript to confirm the accuracy of the data. We received responses from eight of the 27 (30%) with varying degrees of requested modification to our description of their approach to adjudicating abstracts.

### Theoretical Portion of the Project

For the theoretical portion of the project, we worked with thought leaders to enunciate the criteria deemed relevant for the abstract selection process. We divided these criteria into value-based criteria and empirical criteria. We discuss the relative importance of value-based vs. empirical scoring systems.

### Development of a Scoring System for the Society for Academic Emergency Medicine

We designed a scoring system that incorporated all value-based criteria, and report the criteria and the scoring system here. We also describe empirical criteria, though we have not yet applied them to the new scoring system. This effort was led by the Scientific Subcommittee of the Program Committee of the Society for Academic Emergency Medicine. In addition, various thought leaders who were prominent published researchers in the field and had previously provided feedback about the society’s scoring processes also reviewed the developing criteria and provided their input. The end result was an informal consensus-building process.

## RESULTS

### Results of the Survey Portion of the Project

We surveyed 32 medical societies, and obtained data on 28 (response rate 88%). We excluded one of these societies because abstract presentations were not a part of their annual meeting, leaving 27 for analysis.

Table 1 displays the survey results. A minority of societies publish their scoring systems, with only 2 (7%) reporting that their scoring systems were available to submitters. In two cases (the American Heart Association’s Scientific Sessions, and the American Diabetes Association’s Scientific Sessions), the explicit scoring system are not made available, but submitters are informed regarding the domains used to evaluate their submission. The American Heart Association’s Scientific Sessions uses four evaluation domains: scientific merit, organization, presentation, and technical quality. The American Diabetes Association’s Scientific Sessions inform submitters that “originality of work, adequacy of data, and clarity of exposition” are evaluated during the selection process.

Reviews were blinded in most, but not all, societies (21 of 27, or 78%). In fact, we were interested to learn that the submitters’ reputation is an explicit criterion for one society, which requested anonymity.

The number of reviewers per abstract varied, but all reported that each abstract is reviewed by ≥3 reviewers. The American Thoracic Society had the largest number of reviewers per abstract, ranging from five to 15. All but one of the societies asked reviewers to recuse themselves for conflict of interest (96%).

Regarding how scores are created, all but three of our respondents provided information on this. Of the 24 providing information on this topic, most (21, or 88%) reported using a single gestalt score. This ranged from a simple accept/reject vote (as with the Society for Neuroscience), to a 10-point scale (as with the American College of Cardiology).

The remaining three (12%) used a final score created from pooled domain-specific sub-scores. Some societies had different scoring systems for different areas of research (e.g. American Public Health Association), while other societies used a single scoring system for all areas of research (e.g. American Academy of Family Physicians). The scoring system of the American Public Health Association exemplified more complex approaches, and is summarized in Figure. The scoring system of the American Academy of Family Physicians exemplified a simple approach to creating a combined score from pooled domain-specific scores; they scored the following criteria and assigned them a score from 1 to 5: relevance to family medicine, originality/innovative nature of project or question, statement of purpose/goals, project description, evidence-based nature of content, validity of conclusions, and the impact on future work.

### Results of the Theoretical Portion of the Project

In the theoretical portion of this project, we enumerated criteria that could be used to rank abstracts, and we explored other aspects of the abstract selection process. This theoretical work involved discussions among stakeholders. We enunciated potential criteria for abstract selection, and these are listed in Table 2, which divides them into values-based criteria and empirical criteria. Table 3 presents additional considerations that are relevant to the process.

### Development of a Scoring System for the Society for Academic Emergency Medicine

Our consensus led to prioritization of the following values: transparency, fairness, practicality, reviewer qualification, and objectivity. Regarding the other two values listed in Table 2, ease of use and depth, we considered these to be functional opposites, which had to be balanced. In developing our criteria, we had to operationalize our chosen values. We operationalized transparency as public availability of the scoring system and its rationale; hence the present publication.

We operationalized fairness in two ways. First, we felt that reviewers should be blinded so that submissions would be judged on their merits, not based on fame or favoritism. This choice would also prevent bias against more-junior investigators, who are more often unknown to reviewers. Second, we felt that all methodological approaches should be valued equally. Thus, for example, randomized trials should not be given precedence over bench research or qualitative studies. Equal valuation of all methodological approaches was an easy value to enunciate but less easy to enforce with individual reviewers. We used two approaches to cultivate this aspect of fairness. First, we stated this value explicitly in our instructions to reviewers. Second, we developed the scoring criteria with a strategy that explicitly guided reviewers to assign ratings based on merits of the work, not choice of methods (Appendix).

We operationalized practicality by considering the context of the abstract scoring process. Many abstracts must be scored, and then, the accepted abstracts must be published. To facilitate the scoring of many abstracts, we designed a single scoring system that could be applied to any abstract by a qualified reviewer (in contrast to more complex systems, such as that shown in Figure). We also kept the scoring system fairly concise. As another effort toward practicality, we included a specific rating for publication readiness, which considers such things as clarity, grammar, and punctuation. The practical value of this for the society is obvious: after acceptance, each abstract must be converted into a publishable piece, and time is saved by starting with a good product.

We operationalized reviewer qualification by determining that each reviewer should have a reasonable degree of training or experience in medical research. This, in turn, was operationalized as having been first author of at least two peer-reviewed research papers, or having a non-clinical postgraduate degree such as MPH or PhD.

We operationalized the counterbalancing values of ease of use vs. depth by creating a scoring system that was capable of evaluating several dimensions of each abstract (i.e. domains), but was not unduly cumbersome to use. We strove to limit the scoring system to a one-page document.

Operationalization of the concept of objectivity was perhaps most challenging. We felt that a rating system based on a single gestalt evaluation would be entirely subjective. In contrast, a score based on pooled domain-specific scores that were created with explicit ground rules would be relatively objective. Our literature review suggested that objective scoring systems demonstrate greater inter-rater reliability, although the extent to which inter-rater reliability is a goal in and of itself is debated below.^11^–^12^

Having arrived at these values and their operationalization, we set out to create a scoring system, and we now discuss how we reduced these ideas to practice in the creation of an actual document. An integral part of the scoring system was an introduction, which made the values explicit, and was directed at submitters and reviewers alike. The final result is shown in Appendix. The significance of this is that the scoring system is not presented in isolation, but instead comes with an enunciation of values and basic ground rules. By referencing the enhancing the quality and transparency of health research (EQUATOR) network (bottom of first page), it provides an avenue for further self-directed learning. Another noteworthy feature of the first page of this document is the explicit statement that some abstracts may be triaged for no further review.

The first characteristic of this document is its transparency. It begins by enunciating the values underlying the scoring process, and explicitly stating its goals. Next, it describes in detail the administrative process used to get the abstracts scored. It states explicitly what the criteria are for reviewer qualification.

Returning to the value of practicality discussed above, this document then moves on to explain the requirements of the target journal, *Academic Emergency Medicine*. Knowing the rules ahead of time makes the job easier for the submitters, and saves time for journal staff later. It also makes the abstracts more stylistically similar and thus easier for reviewers to adjudicate *en masse*.

The second page of the document provides the scoring system itself. It asks reviewers to assign zero, one, or two points for each of seven domains. This is largely self-explanatory but certain features merit emphasis. Each domain includes a title, followed by an explanation of what is most highly valued in the domain. This is followed by text that illustrates how a reviewer would arrive at a particular score. The wording of all of these segments is designed to be equally applicable to all research methodologies. The validity criterion goes farther, by providing not only general criteria that apply to all abstracts (in the first column on the far left), but also specific examples of how to score various types of research presentations. The statistics and scope scores are not applicable to certain study designs, and this is acknowledged and a “not applicable” option is provided. The final score is a proportion calculated from the points assigned divided by the maximum number of points. The denominator varies according to whether one or two “not applicable” selections are made.

The importance of the scope score may not be as self-evident as the other criteria. A large-scope study is one that has a large sample drawn from multiple locations. Our inclusion of a scope criterion means that a perfectly-designed and perfectly-executed single-center study will score lower than an equivalent multicenter study. Our decision to include this reflects our belief that large-scope studies have already been legitimized by the large funding streams and multiple involved parties necessary to make them happen, and are more likely to be published in the peer-reviewed literature. However, it is important to emphasize that the scope score would not trump the other scores: a large-scope study that was ill-conceived and invalid would still be outscored by a small-scope study of higher quality.

The last criterion is publication readiness. This reflects our value of practicality in developing the scoring system. Abstracts that are sloppy or contain many bizarre abbreviations will be penalized relative to their more-professional counterparts. As mentioned with regard to scope, this score would not trump other scores, and thus it functions as a discriminator at the margin, not an overall test of worth.

## DISCUSSION

No prior research has described how science is selected at medical society meetings across the U.S. We surveyed a convenience sample survey of large U.S. medical conferences, and found that most medical conferences choose abstracts for presentation based on a process that is confidential, is subjective, and is based on a single gestalt rating.

While we conducted this survey, we also developed a theoretical framework for understanding how a medical society might decide how to rank and choose abstracts. Inter-rater reliability is a tempting metric for a scoring system due to its simplicity and objectivity. But this is deceptive because empirical criteria, such as inter-rater reliability, may be superficially relevant as well as prohibitively expensive to collect. More importantly, some empirical criteria may actually conflict with values. For example, it can be argued that reviewers will have different opinions regarding parameters like “importance of the science,” and that thus scoring systems eliciting homogenous reviews would betray plurality of opinion. Similarly, normality of score distribution sounds attractive, but the reality may be that there are many poor-quality abstracts and few good ones, or some other non-normal distribution. Thus, over-emphasis on empirical/objective criteria can be naïve. Table 3 highlights related concerns. The first two points address the extent to which societies rely on an “honor system.” We are aware of no society that routinely audits submissions for prior publication or reviewers for conflicts of interest. While this is understandable based on the difficulty of the proposition, we suggest that with the current modernization of computer technology and library science, cost-effective solutions might not be as far away as we think.

The third point addresses whether an abstract can face summary rejection, i.e. be “triaged.” This use of the term “triage” derives from the National Institutes of Health, which scores some submitted grants but rejects some forthwith, in a process known colloquially as “triaging.” Why would summary rejection be a desirable option? The simplest case would be an abstract that was submitted to the wrong conference. For example, a researcher might submit an abstract relating to the physics underlying magnetic resonance imaging (MRI) technology to an emergency medicine conference, thinking that MRI is part of the scope of emergency care. This type of misunderstanding regarding what is appropriate for the conference is likely to become more common as more and more international research is submitted to U.S. conferences. It would be unfortunate for 3 or more reviewers to spend time puzzling over such an abstract, and trying to score it.

The fourth point raised in Table 3 is one we did not address in our survey: whether two-way communication between submitters and reviewers is allowed. For example, most peer-reviewed journals have administrative staff who will notify submitters when a table is missing or formatting is incorrect. In contrast, our sense is that most medical conferences send submitted abstracts directly to the reviewers. In our proposed system, we have planned for the ability of a reviewer to return an abstract to the submitter when a remediable defect is identified.

The next point raised in Table 3 is whether submitters get any feedback. We did not include this question in our survey, but we assume most societies take the same stance on this issue as we do: there are simply too many abstracts to evaluate and there would be no practical way to provide constructive feedback to so many submitters. The last point in Table 3 is very mundane, but important. When should a medical society evaluate its selection process for scientific presentations at its meeting? Societies that value simplicity above all else can and will continue to use simple “yes/no” votes to select their abstracts. Other, more complex systems may have less longevity.

Abstract scoring does not take place in isolation, but rather is embedded in a context of practical consideration and values. We developed a system for scoring abstracts that is transparent, relatively objective, and based on domain-specific criteria (Appendix). We provide the scoring system to submitters and reviewers alike, and introduce it with an explicit enunciation of the underlying values and expectations.

## LIMITATIONS

The greatest difficulty underlying this discussion is the tension between values-based vs. empirical criteria. Given that our goal is to provide an ordered ranking of objects in a scientific domain, there is an obvious pull to use objective, scientific criteria. The most obvious objective empirical criterion to use to evaluate rating systems is inter-rater reliability, and this has been used previously to evaluate abstract scoring systems. 3–7 Inter-rater reliability is a standard metric in any medical research involving ratings. However, for the present purpose, its desirability as a criterion is mitigated by two considerations. The first is cost. Developing two or more systems and comparing them across multiple raters would be a significant research project in its own right. And where would this lead? After the study, would the documented inter-rater reliability be “good enough?” Would this change over time? Establishing and monitoring inter-rater reliability would be an expensive project and one with no clear endpoint. The second consideration is desirability. As discussed above, we strove to make our new scoring system objective, but it would be wrong not to acknowledge that it retains substantial subjectivity. It remains inevitable that criteria such as “importance” will be judged differently by different raters, and therefore inter-rater consistency would not be expected or desired. The dilemma raised is one of plurality versus homogeneity. We remain unsure as to whether this dilemma can be resolved with finality. Nevertheless, we would be interested to know more about the empirical performance characteristics of our scoring system, such as inter-rater reliability, normality of scores, and generalizability to other disciplines. The scope of the present effort was limited and these questions will have to be pursued in future projects. Furthermore, we acknowledge that there is, and can be, no objective gold standard for what is the best way to select abstracts for presentation. Part of the process is the simple appearance of fairness and objectivity, and for this reason we chose a system that was based upon scores within explicit domains, instead of choosing a gestalt system.

A theoretical limitation is that comparing societies from disparate medical specialties, ranging from nephrology to psychiatry, might be seen as arbitrary. However, in order to have a reasonably large sample of societies, it was necessary not to restrict our convenience sample to one specialty or a narrow group of similar specialties. Also, it bears mentioning that we were not studying the content of the societies’ research, but rather the method by which the research was adjudicated.

## CONCLUSION

We surveyed a convenience sample of large U.S. medical conferences, and found that most do not disclose their criteria to submitters. Most use a single subjective gestalt rating. Laudably, most valued avoidance of favoritism, although reputation was considered a relevant criterion by one society. We developed a scoring system that is transparent, anonymous, and user-friendly. Its objectivity is bolstered by its use of guided domain-specific scoring criteria. Practicality is maximized by clarity of ground rules, provision of a summary rejection procedure, and explicit information about what constitutes a publication-ready abstract.

## Supplementary Information



## Figures and Tables

**Figure:**
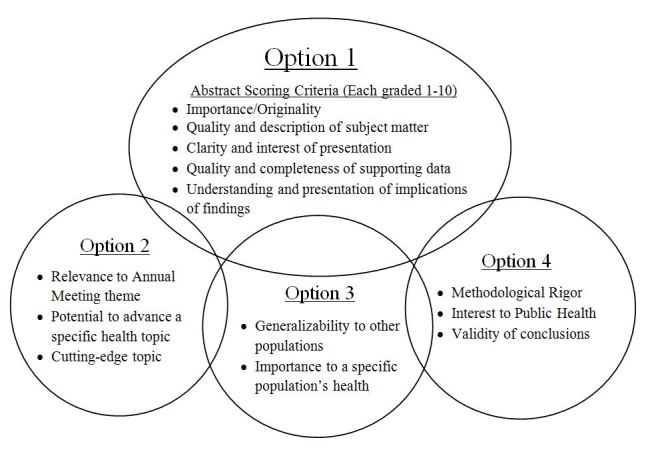
American Public Health Association annual meeting abstract scoring system.

**Table 1:** Abstract selection by medical and scientific societies.

Conference	Scoring system publicly available?	Reviewers blinded?	Number of reviewers/abstract	Recusal for conflict of interest?	Pooled domains or single gestalt?
American Academy of Dermatology	Yes	Yes	≥4	Yes	Single gestalt
American Academy of Family Physicians	No	Yes	8	Yes	Pooled domains
American Academy of Ophthalmology*	No	Yes	≥8	Yes	Single gestalt
American Academy of Pediatrics: Section on Emergency Medicine	No	Yes	5	Yes	Single gestalt
American Academy of Pediatrics: Section on Hospital Medicine	No	Yes	12	Yes	Single gestalt
American Association for Cancer Research Annual Meeting	No	No	4–5	Yes	Single gestalt
American Association of Neurological Surgeons	No	Yes	≥5	Yes	Single gestalt
American College of Cardiology	No	Yes	≥6	Yes	Single gestalt
American College of Emergency Physicians	No	Yes	≥3	Yes	Single gestalt
American College of Rheumatology	No	Yes	Did not disclose	Yes	Did not disclose
American Diabetes Association	No	Yes	6–7	Yes	Single gestalt
American Heart Association: International Stroke Conference	Yes	Yes	≥8	Yes	Single gestalt
American Heart Association: Scientific Sessions	No	Yes	8–10	Yes	Did not disclose
American Psychiatric Association	No	No	≥3	Yes	Single gestalt
American Public Health Association	No	Yes	≥3	Yes	Pooled domains
American Society of Anesthesiologists	No	No	3–4	Yes	Single gestalt
American Society of Clinical Oncology	No	Yes	4–11	Yes	Did not disclose
American Society of Hematology	No	Yes	6	Yes	Single gestalt
American Society of Nephrology - Renal Week	No	Yes	4	Yes	Single gestalt
American Speech-Language-Hearing Association	No	No	≥3	Yes	Single gestalt
American Thoracic Society - International Conference	No	Yes	5–15	No	Single gestalt
American Urological Association	No	Yes	3–5	Yes	Single gestalt
Digestive Disease Week (AGA, AASLD, ASGE, SSAT)	No	Yes	4.5	Yes	Single gestalt
Heart Rhythm Society - Scientific Session	No	Yes	≥3	Yes	Single gestalt
Infectious Diseases Society of America	No	No	3–5	Yes	Single gestalt
Radiological Society of North America**	No	Yes	≥3	Yes	Pooled domains
Society for Neuroscience	No	No	4–6	Yes	Single gestalt

*AGA*, American Gastroenterological Association; *AASLD*, American Association for the Study of Liver Diseases; *ASGE*, American Society for Gastrointestinal Endoscopy; *SSAT*, Society for Surgery of the Alimentary Tract

* The American Academy of Ophthalmology has a two-staged review. Five general reviewers conduct the first review, and the second review has 3 subspecialty reviewers that take the first-round score into account and make a final judgment in a conference call.

** The Radiological Society of North America allows the chairperson of each subspecialty to interpret his or her own grading scale. In other words, this society has a gestalt scoring system with the option to create a more nuanced, pooled domains system. The chairperson personalizes the scoring system based on the criteria and themes that are important to the subspecialty in any given year.

**Table 2:** Criteria for the creation of scoring systems.

Value-based criteria	Empirical criteria
Transparency	Inter-rater reliability
Ability to accommodate plurality of opinion	Normality of score distribution
Fairness (no favoritism; equal consideration for all methodologies)	Time required to assign a score
Practicality	Generalizability of score from reviewers to conference attendees
Reviewer qualification	Popularity
Objectivity	Predictive value for an abstract resulting in a peer-reviewed publication
Ease of use	Predictive value for an abstract resulting in a grant
Depth	

**Table 3:** Other considerations in abstract presentation.

Additional Considerations
Does the society do anything to confirm that abstracts have not been presented previously?
Does the society do anything to seek undeclared conflicts of interest among reviewers?
Is there an initial system for “triaging” abstracts that do not require formal scoring?
Can reviewers return an abstract to the submitter for correction?
Is there a formal process for feedback about the scoring system?
Is the scoring system reviewed and updated according to any pre-specified schedule?

## References

[1] Scherer RW, Dickersin K, Langenberg P (1994). Full publication of results initially presented in abstracts. A meta-analysis. JAMA.

[2] Scherer RW, Langenberg P, von Elm E (2007). Full publication of results initially presented in abstracts. The Cochrane Database Syst Rev.

[3] Timmer A, Sutherland LR, Hilsden RJ (2003). Development and evaluation of a quality score for abstracts. BMC Med Res Methodol.

[4] Poolman RW, Keijser LC, de Waal Malefijt MC (2007). Reviewer agreement in scoring 419 abstracts for scientific orthopedics meetings. Acta orthop.

[5] van der Steen LP, Hage JJ, Kon M (2003). Reliability of a structured method of selecting abstracts for a plastic surgical scientific meeting. Plast Reconstr Surg.

[6] Ector H, Aubert A, Stroobandt R (1995). Review of the reviewer. Pacing Clin Electrophysiol: PACE.

[7] Rubin HR, Redelmeier DA, Wu AW (1993). How reliable is peer review of scientific abstracts? Looking back at the 1991 Annual Meeting of the Society of General Internal Medicine. J Gen Intern Med.

[8] van der Steen LP, Hage JJ, Kon M (2004). Validity of a structured method of selecting abstracts for a plastic surgical scientific meeting. Plast Reconstr Surg.

[9] Smith J, Nixon R, Bueschen AJ (2002). Impact of blinded versus unblinded abstract review on scientific program content. J Urol.

[10] http://c.ymcdn.com/sites/www.hcea.org/resource/resmgr/docs/june_2014_top_50_total_atten.pdf.

[11] Rowe BH, Strome TL, Spooner C (2006). Reviewer agreement trends from four years of electronic submissions of conference abstract. BMC Med Res Methodol.

[12] Montgomery AA, Graham A, Evans PH (2002). Inter-rater agreement in the scoring of abstracts submitted to a primary care research conference. BMC Health Serv Res.

