# Supplementary material for: How do Medical Societies Select Science for Conference Presentation? How Should They?
Source: West J Emerg Med. 2015 Jul 2;16(4):543–50. doi: 10.5811/westjem.2015.5.25518 (PMC4530912; doi:10.5811/westjem.2015.5.25518)
Supplement: Supplementary file 1 [file wjem-16-543-s001.pdf]

Submitting your abstract to the SAEM Annual Meeting

SAEM
Society for Academic Emergency Medicine

SCIENTIFIC ABSTRACT SCORING SYSTEM

SUBMISSION GUIDELINES

In choosing abstracts for our annual meeting, SAEM's goals are logic, fairness, and transparency. Abstracts are scored according to their scientific quality, medical importance, and publication readiness. We do not believe one form of research is inherently better than another (e.g., clinical trials vs. health services research vs. qualitative studies).

We are pleased to announce that, as of 2015, we will publish our abstract scoring criteria. We think this will help new researchers produce better abstracts, and we think junior and senior researchers alike will appreciate the transparency. The publication readiness score is an important new addition. Every accepted abstract is published in *Academic Emergency Medicine* (and indexed in PubMed), and well-written abstracts make better publications in the journal.

We use a two-stage process for scoring abstracts. First, a primary reviewer evaluates each abstract, and may send it back to the author if something is missing, or may triage the abstract for no further review, if major problems are identified. If the abstract passes this initial review, it moves on to full review. Each fully-reviewed abstract is scored by at least 3 qualified reviewers. Effective 2015, SAEM will require that all reviewers have first-authored two or more peer-reviewed original research articles or have an MPH, PhD, or equivalent non-clinical healthcare degree.

Remember, scoring is a judgment call. You are welcome to use the criteria to score your own abstract, but this won't change how the reviewers called the play at game-time.

The SAEM Annual Meeting is *your* meeting! We hope you find it helpful to understand the selection process as you prepare your abstract, and we hope this helps new researchers grow.

Here are some tips to help you save on word count, and to get your abstract ready for publication in *Academic Emergency Medicine:*

General

- Make sure your name, degrees, and affiliations are quoted consistently on every abstract that lists you as an author. Double check.
- If one research project is split into two or more abstracts, they may be rejected for "data splitting."
- Have a colleague critique and proofread the abstract each time you revise it, especially if you do not usually write in English.
- You do not have to mention IRB or animal committee approval in your abstract, because you will attest to that during submission.
- Do not include bibliographic references in your submitted abstract. (You may do so in your poster or presentation.)
- Do not use fonts smaller than 10 point.

Figures and Tables

- Be sure your figure or table has uploaded correctly to the system before you hit the submit button. Double check!
- Figures can use color. Figure/table width is limited to 8.6 cm. Include the author's name in the title (e.g. "Jones Figure 1").
- Be sure there is a "callout" for each figure/table in the text of the abstract (e.g. "(see Jones Figure 1)").

Statistics and Analytical Software

- Use this format for confidence intervals: (95%CI 2.0-2.7). Use "to" if there is a negative number, as in (95%CI –2.0 to 2.7).
- You do not have to say what statistical software you used. Save that for the manuscript.
- Mention your statistical alpha only if it is something other than 0.05.

Grammar and Style

- The following standard abbreviations will be shown for all abstracts, so you can use them without defining them: 95% CI, AAAEM, AAEM, ACEP, AEUS, AGEM, AWAEM ACGME, ADIEM, AIDS, ASA, AUC, BP, bpm, CBC, CDEM, CORD, CPR, CT, CXR, dBP, ECG, ED, EM, EMS, FDA, GEMA, HIV, INR, IQR, IV, mmHg, MRI, NIH, PGY, ROC, SAEM, sBP, SIM, SD (when used as standard deviation after a mean), tPA, U/S.
- Do not use other new or uncommon abbreviations unless it is unavoidable. Do not define abbreviations/acronyms in the title.
- Never use an abbreviation in place of a single English word; for example, never use "pts" in place of "patients."
- Only capitalize the first word of each sentence, acronyms, and proper names. It is not necessary to capitalize "emergency medicine."
- In sentences, do not use symbols instead of words (like & instead of "and"). Using symbols in the usual way (25% or $25) is fine.
- There should be a space between a number and its units. For example, 80 mmHg is correct, but 80mmHg is not.
- Use a leading zero for all decimal figures; for example, 0.2.
- Do not use underlined < and > and + symbols. Instead use the "Insert / Symbol" function to show the real ≤ and ≥ and ± symbols. Use the "Insert / Symbol" function for the ° (degree) symbol, rather than a superscript o.

- **Anyone seeking more guidance about how research should be reported can refer to http://www.equator-network.org/**

SAEM Society for Academic Emergency Medicine

SCIENTIFIC ABSTRACT SCORING SYSTEM

SCORING CRITERIA

*Reviewers, follow criteria **literally**, and score each domain **independently**.*

***CLARITY OF OBJECTIVES*—Reviewers prioritize studies with clear objectives (whether descriptive or hypothesis-testing).**

2 Well thought out study objectives, or clearly stated and testable hypothesis.
1 Stated objectives were poorly chosen, or stated hypothesis was difficult to test.
0 No clear objectives or hypothesis, or not relevant to emergency medicine.

***CHOICE OF APPROACH*—Reviewers prioritize studies that use the right research methods for the scientific question.**

2 Chosen study design was the best feasible method for testing the stated hypothesis/objectives (*i.e.* a robust design).
1 Chosen study design was sub-optimal but did test the stated hypothesis/objectives (*i.e.* an acceptable design).
0 Design did not test stated hypothesis/objectives, or not relevant to emergency medicine.

***VALIDITY*—Were the right outcomes measured in the right way? Were potential confounders managed well? Is the story logical?**

	Scoring Criteria	*Specific Examples (abstract not required to fit in one of these specific categories – see general Scoring Criteria at left)*					
		Clinical Trial	*Observational Study*	*Survey*	*Laboratory*	*Qualitative Research*	*Meta-analysis*
2	Well-controlled, well protected from bias, and presented very clearly	Appropriately randomized, blinded, and controlled	Excellent control of bias and confounding. Clean data acquisition	Few non-respondents, sampling bias unlikely, clear constructs, robust analysis	Excellent methods, and experimental control, can replicate	Analytic framework, coding, and interview guides clear. Session notes and recordings.	Exhaustive search and selection criteria, good heterogeneity control
1	Protection against bias, experimental control, and presentation satisfactory	Randomized for main outcome, vulnerable to bias or poor blinding	Bias/confounding controlled with some shortcomings; data acquisition reasonable	Response rate adequate but not impressive, valid constructs, clear analyses	Adequate methods and experimental controls	Analytic framework, coding, or guides not perfect, session notes or recordings	Adequate search and selection criteria, or fair heterogeneity control
0	Poorly controlled and vulnerable to bias, vague, confusing, or illogical	Not randomized for main outcome, or faulty randomization	Unclear methods, vulnerable to bias/confounding, or invalid data acquisition	Flawed logic, low response rate, or respondents may differ from non-respondents	Methods invalid, poor experimental control, or cannot replicate	Analytic framework, coding, or guides not specified, or poor session documentation	Unclear search or selection criteria, or inappropriate pooling

***STATISTICS*—Reviewers prioritize studies that use statistics correctly.**

X Skip this question because statistics are not applicable – this is a study type that should not be scored based on inferential statistics (e.g. qualitative study).
2 Statistical methods and conclusions are correct. The reader has a clear understanding of the possibility of Type I and Type II error.
1 Statistical methods and conclusions are technically flawed, but the reader is able to understand the possibility of Type I and Type II error. Conclusions are accurate.
0 The reader is not given a clear understanding of the relative importance of *variation targeted for measurement* versus *random variation* (i.e. signal vs. noise).

***SCOPE*—Reviewers prioritize large multicenter studies over small single-center studies.**

X Skip this question because this is a basic science study or another study type for which scope is clearly not relevant.
2 Large, multicenter study likely to be published in major journal. For example, randomized trial with >5 sites and >200 subjects, or large multicenter educational study.
1 Moderate-sized study. For example, a randomized trial of 100 subjects at 3 EDs, or a process improvement study that includes 5 EDs in different states.
0 Small N in a study of a common disease. For example, a clinical trial of 50 subjects at one center, or a qualitative study with 8 participants.

***IMPORTANCE OF TOPIC*—Reviewers prioritize topics of major importance to large numbers of emergency medicine researchers or clinicians. Reward innovation.**

2 This topic, or its foreseeable progeny, is relevant to *every emergency physician*, or is highly innovative.
1 This is an important topic that will lead to information of interest to *many or most emergency physicians*, including those who do not study this topic.
0 This topic is only of interest to the small group of people who study it, and is unlikely to result in important knowledge.

***PUBLICATION READINESS*—Does this abstract reflect high quality writing and attention to detail?**

2 Perfect grammar, no errors, very clear expression of ideas. Conforms perfectly to our SAEM submission guidelines.
1 Generally well written, but leaves room for confusion on some concepts or has one or two errors.
0 Poorly written. Hard to understand, idiosyncratic phrasing, or awkward abbreviations.
